# Role of Extrachromosomal Histone H2B on Recognition of DNA Viruses and Cell Damage

**DOI:** 10.3389/fgene.2013.00091

**Published:** 2013-05-23

**Authors:** Kouji Kobiyama, Akira Kawashima, Nao Jounai, Fumihiko Takeshita, Ken J. Ishii, Tetsuhide Ito, Koichi Suzuki

**Affiliations:** ^1^Laboratory of Adjuvant Innovation, National Institute of Biomedical InnovationIbaraki, Osaka, Japan; ^2^Laboratory of Vaccine Science, Immunology Frontier Research Center, World Premier International Research Center, Osaka UniversitySuita, Osaka, Japan; ^3^Laboratory of Molecular Diagnostics, Department of Mycobacteriology, Leprosy Research Center, National Institute of Infectious DiseasesTokyo, Japan; ^4^Division of Bioimaging Sciences, Center for Molecular Medicine, Jichi Medical UniversityShimotsuke, Japan; ^5^Department of Medicine and Bioregulatory Science, Kyushu UniversityFukuoka, Japan

**Keywords:** DNA sensor, extrachromosomal histone, virus infection, DNA damage, epigenetic modifications

## Abstract

Histones are essential components of chromatin structure, and histone modification plays an important role in various cellular functions including transcription, gene silencing, and immunity. Histones also play distinct roles in extrachromosomal settings. Extrachromosomal histone H2B acts as a cytosolic sensor to detect double-stranded DNA (dsDNA) fragments derived from infectious agents or damaged cells to activate innate and acquired immune responses in various cell types. It also physically interacts with interferon (IFN)-β promoter stimulator 1 (IPS-1), an essential adaptor molecule that activates innate immunity, through COOH-terminal importin 9-related adaptor organizing histone H2B and IPS-1 (CIAO), resulting in a distinct signaling complex that induces dsDNA-induced type I IFN production. Such a molecular platform acts as a cellular sensor to recognize aberrant dsDNA in cases of viral infection and cell damage. This mechanism may also play roles in autoimmunity, transplantation rejection, gene-mediated vaccines, and other therapeutic applications.

## Introduction

Epigenetic modifications of histones, the primary protein component of chromatin, contribute to diverse homeostatic cellular activities such as transcriptional regulation, chromosome condensation (mitosis), apoptosis, and DNA repair (Bradbury, 1992; Koshland and Strunnikov, 1996; Rogakou et al., 2000; Fernandez-Capetillo et al., 2004). Histones are divided into two groups based on their principal functions. Histones H2A, H2B, H3, and H4 are known as the core histones. Two of each core histone form the histone octamer, which genomic DNA wraps around to form a nucleosome (Luger et al., 1997). Histone H1, the linker histone, binds and rearranges the DNA between nucleosome units (linker DNA) to assist chromatin compaction. Interestingly, histones are present in cytosol (Kobiyama et al., 2010) as well as in the nucleus, mitochondria (Konishi et al., 2003), and cell surface (Radic et al., 2004), particularly during viral infections, apoptosis, and cell damage. Histone H2B transits in and out of the nucleosome more rapidly than other core histones, such as H3 and H4. Thus, about 3% of total H2B is exchanged within 6 min (*t*_1/2_), ∼40% within 130 min, and ∼50% by 8.5 h (Kimura, 2005). Histones have microbicidal activity in neutrophil extracellular traps (NETs), which are composed of DNA, elastase, and histones. Treatment of NETs with histone neutralizing antibodies resulted in reduced bactericidal activity against species such as *Shigella flexneri* and *Staphylococus aureus* (Brinkmann et al., 2004). Thus, these “extrachromosomal” histones play important roles in physiological conditions, including innate and adaptive immune responses. We recently reported that extrachromosomal histone H2B is involved in the recognition of cytosolic double-stranded DNA (dsDNA) generated by DNA viruses (non-self) and genomic DNA from damaged cells (self) (Kobiyama et al., 2010; Kawashima et al., 2011a).

## DNA-Mediated Immune Response

In 1963, Alick Isaacs found that nucleic acids, both DNA and RNA, strongly induce innate immune responses, such as type I interferon (IFN) production (Isaacs et al., 1963; Rotem et al., 1963). Although this finding generated a great deal of excitement in the field of immunology at that time, it was forgotten or largely ignored until it was shown that unmethylated CpG DNA stimulates immune cells to produce cytokines (Tokunaga et al., 1984; Krieg et al., 1995). As a result, most immunologists presumed that unmethylated CpG DNA was the essential element within self and non-self DNA that activated innate immunity. Toll-like receptor 9 (TLR9) was subsequently identified as a cellular receptor for unmethylated CpG DNA in the activation of innate immune responses in immune cells, such as dendritic cells (DCs), B cells, and macrophages (Hemmi et al., 2000, 2003). In the meantime, dsDNA independent of unmethylated CpG motifs or any other specific sequence was shown to up-regulate the expression of genes related to the immune response (Suzuki et al., 1999). In particular, genomic dsDNA released by injured cells induces maturation of antigen presenting cells and adaptive immune responses (Ishii et al., 2001). Furthermore, TLR9-dependent and -independent IFN-α production is induced in response to herpes simplex virus-1 (HSV-1) infection (Hochrein et al., 2004). It was later confirmed that the right-handed helical structure (B-form) of DNA is the component responsible for induction of robust type I IFNs in both immune and non-immune cells through TLR9-independent recognition and signaling cascades (Ishii et al., 2006; Stetson and Medzhitov, 2006).

The harmful effects of aberrant DNA have been shown in relation to the function of enzymes that digest DNA (DNases). Thus, hepatic macrophages in DNase II-deficient mice failed to digest DNA from engulfed nuclei of erythroblasts and exhibited robust production of type I IFN, which resulted in severe anemia and development of rheumatoid arthritis (RA)-like symptoms in a TLR9-independent manner (Yoshida et al., 2005; Kawane et al., 2006). DNase I and DNase III knockout mice showed systemic lupus erythematosus (SLE)-like symptoms and inflammatory myocarditis, respectively (Napirei et al., 2000; Yasutomo et al., 2001; Morita et al., 2004). The functional mutations of DNase I and DNase III in humans have also been associated with several autoimmune disorders, such as SLE (Yasutomo et al., 2001; Lee-Kirsch et al., 2007b), Aicardi–Goutieres syndrome (Crow et al., 2006), familial chilblain lupus (Lee-Kirsch et al., 2007a), and retinal vasculopathy with cerebral leukodystrophy (Richards et al., 2007). Thus, DNA-induced immune responses are involved in the prevention of both microbial infection and autoimmune responses. These findings also suggest that normal cells are equipped with innate machinery that senses and removes aberrant genomic DNA fragments before they produce pathological effects.

## Cytosolic Sensors for DNA Fragments and Their Metabolites

Several proteins have been identified as DNA sensors that recognize aberrant cytosolic DNA fragments and their metabolites. These sensors are involved in the elimination of invasive pathogens and the induction of inflammation. In most cases, recognition of cytosolic DNA by these sensors results in induction of innate immune responses through several key proteins such as stimulator of interferon genes (STING) and TANK-binding kinase 1 (TBK1) (Ishii et al., 2006; Ishikawa and Barber, 2008). STING and TBK1 are also essential factors in the immunogenicity of plasmid DNA vaccines (Ishii et al., 2008; Ishikawa et al., 2009). The underlying mechanisms for the immunological advantages of DNA vaccines have not been fully elucidated. However, it has been suggested that the detection of the double-stranded structure of plasmid DNA by cytosolic DNA sensors contributes to an enhanced adaptive immune response to the vaccine antigen.

Z-DNA binding protein 1/DNA-dependent activator of IFN-regulatory factors (ZBP-1/DAI) was the first reported cytosolic DNA sensor (Takaoka et al., 2007). ZBP-1/DAI contains two Z-DNA binding domains and a D3 domain, all of which are essential for its activation. Overexpression of ZBP-1/DAI enhanced dsDNA-mediated gene expression and knockdown of ZBP-1/DAI impaired IFN-β production by HSV-1 infection, but not Newcastle disease virus (NDV) infection, in a mouse fibroblast cell line (Takaoka et al., 2007). However, fibroblasts from ZBP-1/DAI deficient mice normally responded to dsDNA, and the mice also showed normal immunogenicity to plasmid DNA vaccinations (Ishii et al., 2008).

In 1993, it was reported that electroporated DNA induces cell death in murine macrophages (Stacey et al., 1993). Recently, absence in melanoma 2 (AIM2) was identified as a cytosolic DNA sensor for activation of the inflammasome, a large multimolecular complex that regulates activation of the enzyme caspase-1, to induce IL-1β production and DNA-induced cell death. AIM2 is a member of the hematopoietic IFN-inducible nuclear protein with a 200-amino-acid repeat (HIN-200) family, which contains a pyrin domain and a DNA-binding HIN-200 domain. AIM2 recognizes cytosolic DNA and interacts with inflammasome-related molecules to induce pyroptosis, a type of programed cell death characterized by activation of caspase-1 and IL-1β production upon inflammatory antimicrobial responses. Deficiency of AIM2 results in an enhancement of susceptibility to bacteria and DNA viruses (Burckstummer et al., 2009; Fernandes-Alnemri et al., 2009; Hornung et al., 2009; Roberts et al., 2009).

Interferon gamma inducible protein 16 (IFI16) is a member of the pyrin and HIN domain-containing (PYHIN) protein family that contains a pyrin domain and two DNA-binding HIN domains. IFI16 directly binds viral DNA in the cytosol and induces IFN-β production through STING (Unterholzner et al., 2010). Small interfering RNA (siRNA) for IFI16 inhibited DNA-induced but not RNA-induced IFN-β production. Knockdown of p204, a mouse ortholog of IFI16, impaired activation of transcription factors and gene inductions upon DNA virus infection.

Although retinoic acid-inducible gene I (RIG-I) was initially identified as a cytosolic RNA receptor, it is also involved in the recognition of cytosolic dsDNA. Thus, knockdown of RIG-I in human hepatocellular carcinoma cell line, HuH-7, attenuated dsDNA-induced type I IFN production. Subsequently, it was shown that poly(dA·dT)·poly(dT·dA) and DNA virus-derived DNAs were converted into 5′-triphosphate RNA by RNA polymerase III to induce RIG-I-mediated type I IFN production. This IFN production induced by intracellular bacteria was abolished by a specific inhibitor of RNA polymerase III, which in turn resulted in a promotion of bacterial growth (Chiu et al., 2009).

High mobility group box protein 1 (HMGB1), initially identified as a non-histone DNA-binding and chromatin-associated protein, is involved in DNA organization and transcriptional regulation (Goodwin et al., 1973; Bustin, 1999). Although most of HMGB1 is localized to the nucleus, HMGB1 acts as an “alarmin” to promote inflammation upon its release from the nucleus during necrosis (Scaffidi et al., 2002). In addition, extracellular HMGB1 is involved in the pathogenesis of autoimmune diseases, as evidenced by the presence of anti-HMGB1 autoantibodies in sera from RA and drug-induced SLE patients (Wittemann et al., 1990; Ayer et al., 1994). The HMGBs (HMGB1, HMGB2, and HMGB3) also bind immunogenic nucleic acids, e.g., virus-derived RNAs and genomic DNAs, and activate innate immune signaling through receptor for advanced glycation and end products (RAGE). In fact, knockdown of HMGBs resulted in a reduction of innate immune responses against immunogenic nucleic acids (Yanai et al., 2009).

In human cells, various types of DNA reportedly induce type III IFNs, especially IFN-λ1 (or interleukin29; IL29). Ku70, whose original functions were reported as DNA repair, V(D)J recombination and telomerase maintenance, was identified as a cytosolic DNA sensor that is responsible for the induction of IFN-λ1 (Zhang et al., 2011a). Knockdown of Ku70 suppressed IFN-λ1 activation in human cells. Whereas other known DNA sensors are involved in type I IFN production, Ku70 is unique in the production of type III IFN upon dsDNA stimulation.

Leucine-rich repeat flightless-interacting protein 1 (LRRFIP1) was initially identified as an RNA-binding protein, but it was eventually recognized as a receptor for both exogenous DNA and RNA (Yang et al., 2010). LRRFIP contains a DNA-binding domain, and is responsible for the production of IFN-β through interaction with β-catenin and recruitment of acetyltransferase p300 in cases of vesicular stomatitis virus (VSV) and *Listeria monocytogenes* infection.

RNA and DNA helicases are members of the DEADbox family, the name of which was derived from one of the conserved amino-acid sequences in the proteins. Members of the DExD/H-box (where x can be any amino acid) helicase superfamily, such as DHX9 and DHX36, were identified as cytosolic CpG DNA sensors for the induction of type I IFN production in plasmacytoid DCs (Kim et al., 2010). Another helicase, DDX41, a member of the DEXDc family, was identified as an intracellular dsDNA sensor that is responsible for type I IFN production in myeloid DCs (Zhang et al., 2011b). After stimulation with dsDNA, DDX41 interacts with STING in the microsome, mitochondria, and mitochondria-associated endoplasmic reticulum membrane fractions. DDX41 also recognizes bacterial second messenger cyclic di-GMP and cyclic di-AMP, and activates type I IFN production by interacting with STING, leading to TBK1-IRF3 activation (Parvatiyar et al., 2012).

DNA transfection or DNA virus infection leads to a production of cyclic GMP-AMP (cGAMP) via the function of cGAMP synthase, cGAS, which belongs to the nucleotidyltransferase family, and an endogenous second messenger to induce innate immune responses. cGAS binds to DNA in the cytoplasm and catalyzes cGAMP synthesis to function as a cytosolic dsDNA sensor that induces type I IFNs (Sun et al., 2013). It was also shown that cGAMP directly interacts with STING to activate IRF3, and knockdown of cGAS results in the suppression of IFN-β production induced by dsDNA transfection or DNA virus infection (Sun et al., 2013).

These studies were performed using different types of cells, synthetic DNAs, bacteria, and viruses as shown in Table 1. Therefore, it should be noted that multiple recognition machineries for sensing cytosolic DNA and DNA metabolites might differ among species and/or cell types.

**Table 1:** **Cytosolic DNA sensors**.

DNA sensor	Localization	Pathogens	Nucleic acid ligand	Reference
ZBP-1/DAI	Cytoplasm	HSV	Poly(dA:dT), ISD	Takaoka et al. (2007)
AIM2	Cytoplasm	VV, MCMV, *L. monocytogenes*, *F. tularensis*	Calf thymus DNA, poly(dA:dT)	Burckstummer et al. (2009), Fernandes-Alnemri et al. (2009), Hornung et al. (2009), Roberts et al. (2009)
IFI16	Cytoplasm	VV, HSV-1	Poly(dA:dT)	Unterholzner et al. (2010)
RNA pol III/RIG-I	Cytoplasm	*L. pneumophila*, AdV, HSV-1, EBV	Poly(dA:dT)	Chiu et al. (2009)
HMGB1	Nucleus, extracellular	VSV, HSV-1	dsDNA, dsRNA, ssDNA, ssRNA	Yanai et al. (2009)
Ku70	Cytoplasm	HIV?	Plasmid DNA	Zhang et al. (2011a)
LRRFIP1	Cytoplasm	*L. monocytogenes*, VSV	Poly(dA:dT)	Yang et al. (2010)
DDX41	Cytoplasm	*L. monocytogenes*, AdV, HSV-1, VV	Poly(dA:dT), c-d-GMP, c-d-AMP	Zhang et al. (2011b), Parvatiyar et al. (2012)
cGAS	Cytoplasm	HSV-1	cGAMP	Sun et al. (2013)
Histone H2B	Nucleus, cytoplasm	HPV, AdV, HIV	Poly(dA:dT), genomic DNA	Kobiyama et al. (2010), Kawashima et al. (2011a)

*HSV, herpes simplex virus; VV, vaccinia virus; MCMV, mouse cytomegalovirus; AdV, adenovirus; EBV, Epstein–Barr virus; VSV, vesicular stomatitis virus; HIV, human immunodeficiency virus; HPV, human papilloma virus; dA:dT, poly(dA-dT)·poly(dT-dA); ISD, immunostimulatory DNA*.

## Extrachromosomal Histone H2B is Involved in DNA Sensing

To identify molecules responsible for cytosolic dsDNA-mediated type I IFN production, we screened a cDNA expression library using HEK293T cells stably transfected with a luciferase gene cassette under an IFN-β promoter. Among >960,000 independent clones examined, a single clone encompassing the histone H2B ORF exhibited a striking enhancement of dsDNA-induced IFN-β promoter activation (Kobiyama et al., 2010). In a separate set of experiments, cellular proteins that bind dsDNA were purified from rat thyroid cell line FRTL-5, cells previously proven to respond well to dsDNA (Suzuki et al., 1999). Protein extracts were passed through ssDNA sepharose and absorbed onto dsDNA sepharose columns before electrospray ionization (ESI)-MS/MS mass spectrometry analysis. Among the molecules identified, histone H2B showed a significantly high MASCOT (probability) score (Kawashima et al., 2011a). Thus, two independent approaches implied that extrachromosomal H2B functionally mediates IFN-β promoter activation in human kidney cells following dsDNA stimulation and physically associates with dsDNA in rat thyroid cells.

Type I IFN production induced by dsDNA was significantly suppressed in HEK293 cells treated with H2B siRNA, but not by those treated with siRNAs for other core histones. Although most histone H2B localizes in the nucleus, it appears to sense DNA in the cytoplasm by interacting with IFN-β promoter stimulator 1 (IPS-1) (Kobiyama et al., 2010), an essential adaptor molecule for signal activation triggered by cytoplasmic dsRNA and single stranded 5′-triphosphate RNA (Kawai et al., 2005; Meylan et al., 2005; Seth et al., 2005; Xu et al., 2005). Human, but not mouse, IPS-1 was involved in the dsDNA-mediated signal transduction (Kumar et al., 2006; Ishii et al., 2008). Therefore, histone H2B interacts with IPS-1 in the cytoplasm following dsDNA stimulation only in human cells.

Yeast two-hybrid screening identified KIAA1192 as a molecule that interacts directly with histone H2B; therefore, it was renamed CIAO (C-terminal importin 9-related adaptor organizing histone H2B and IPS-1) based on its novel role. While high similarities of amino acid sequences were detected between human and mouse H2B (>70.1%) and between human and mouse CIAO (99.2%), the amino acid sequence of IPS-1 was largely different between human and mouse (30.3%). The observed interaction of CIAO and IPS-1 only in human molecules is a possible reflection of this difference in IPS-1 sequence (Kobiyama et al., 2010). These results strongly suggest that there is species-specific involvement of IPS-1 in dsDNA-mediated signaling.

**Figure 1:**
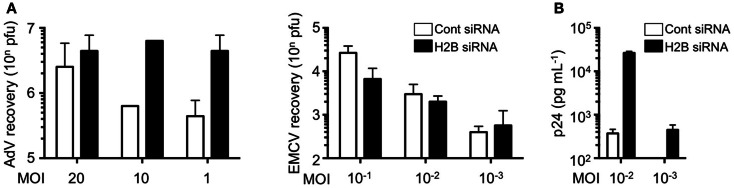
**Histone H2B is a key factor for the suppression of viral replication**. **(A)** HEK293 and HeLa cells were pretreated with control siRNA (Cont siRNA) or histone H2B siRNA (H2B siRNA). The cells were infected with AdV type 5 or EMCV. Twenty-four hours after infection, viral multiplication was determined by a plaque assay. **(B)** Magic 5 cells were pretreated with Cont siRNA or H2B siRNA. The cells were infected with HIV-1 IIIB for 3 h. Seventy-four hours after infection, viral multiplication was determined by HIV-1 p24 ELISA using culture supernatant.

We further examined the role of histone H2B on cell-autonomous antiviral responses. Knockdown of histone H2B suppressed IFN-β production and STAT1 phosphorylation when DNA viruses, in this case modified vaccinia virus Ankara (MVA), were infected (Kobiyama et al., 2010). Multiplication of adenovirus type 5 was significantly enhanced in the H2B knockdown cells, while multiplication of RNA viruses, such as encephalomyocarditis virus (EMCV), was not affected by the presence or absence of histone H2B (Figure 1A). Multiplication of other DNA viruses, such as human papilloma viruses (HPV11 and HPV16) and adenovirus serotype 5, was significantly enhanced in cells to which histone H2B siRNA was transfected. These results suggested that extrachromosomal histone H2B is involved in the sensing of DNA viruses and mediates cell-autonomous antiviral immune responses in human cells. The human immunodeficiency virus (HIV) is a lentivirus, a class of retrovirus, which has two copies of positive single stranded RNA that codes viral genes. Upon infection in target cells, the viral RNA genome is reverse transcribed into dsDNA in the peri-integration complex (PIC). When histone H2B was knocked-down in CCR5-expressing HeLa/CD4^+^ cell clone 1–10 (Magic 5) cells, HIV-1 replication was significantly enhanced (Figure 1B). These data clearly indicate that histone H2B discriminates between foreign DNA and RNA upon viral infection to evoke IPS-1-mediated signaling through association with a novel adaptor protein, CIAO. It has also been suggested that human IPS-1 has evolutionarily gained the potential to transmit dsDNA-initiated, histone H2B-mediated signaling to combat human viruses that produce DNA intermediates within the cell. Whether histone H2B has a role in infection in mice, probably by interacting with molecules other than IPS-1, is currently unknown.

We next examined the involvement of genomic DNA-mediated immune responses in light of a possible role in the triggering of autoimmune disorders. When FRTL-5 thyroid cells were exposed to progressively higher levels of electric pulsing, in the absence of pathogens or immune cells, genomic DNA was released to the cytoplasm, which was associated with activation of the expression of certain genes, such as those encoding type I IFN and chemokines. More importantly, the expression of major histocompatibility complex (MHC) class II molecules and co-stimulatory molecules was also induced in thyroid cells (Suzuki et al., 1999; Kawashima et al., 2011a), suggesting that the autoimmune target cell itself might present autoantigens upon cell damage (Kawashima et al., 2011b). It has been assumed that autoimmune thyroid diseases, such as Graves’s disease and Hashimoto’s thyroiditis, develop by a combination of genetic susceptibility and environmental factors. The data suggested that thyroid cell injury results in the release of genomic DNA fragments into the cytosol, which are recognized by extrachromosomal histone H2B to activate genes involved in both innate and acquired immune responses. Such responses may relate to the development of thyroiditis that in turn may increase the chance to present self-antigens to immune cells and initiate autoimmune reactions. Thus, our findings suggest that extrachromosomal histone H2B acts as a cytosolic DNA sensor for both self and non-self DNA, and that this recognition mechanism may be involved in preventing microbial infections and triggering of autoimmune disorders.

## Epigenetic Modification and Virus Infection

Epigenetic modifications, including histone modifications and chromatin remodeling, regulate cellular processes that require access to genomic DNA. DNA viruses utilize the chromatin-mediated regulation of gene transcription and DNA replication of the host cell (Liang et al., 2009). In the case of herpes viruses, chromatin modulation is a regulatory factor of viral latency and reactivation cycles. Infection of cells with herpes virus results in the deposition of nucleosomes bearing repressive K9 methylation of histone H3 (H3-K9) on the viral genome. Inhibition of lysine-specific demethylase (LSD1) results in an accumulation of repressive chromatin and blockage of viral gene expression (Liang et al., 2009). In the case of HIV-1, histone H3-K9 methyltransferase G9a is responsible for chromatin-mediated HIV-1 transcriptional latency through methylation of H3 (Imai et al., 2010). In addition, K9 methylation of histone H3 is involved in repression of the human cytomegalovirus gene (Ioudinkova et al., 2006). Thus, since viruses utilize the host gene regulation system for their replication, its modification blocks initial gene expression of a DNA virus, including adenovirus (Liang et al., 2013).

Histone H2B can also be modified by acetylation (Schiltz et al., 1999), GlcNAcylation (Fujiki et al., 2011), phosphorylation (Fernandez-Capetillo et al., 2004), sumoylation (Nathan et al., 2006), and ubiquitination (Zhu et al., 2005), but not by citrullination and methylation. Thus, histone H2B acetylation (K12 and K15) is involved in transcriptional activation (Schiltz et al., 1999; Kawasaki et al., 2000), and phosphorylation of histone H2B (S14) is an epigenetic marker of apoptotic cells (Cheung et al., 2003). Deacetylation of K15 is essential for H2B S14 phosphorylation, and inhibition of deacetylation suppresses internucleosomal DNA degradation (Ajiro et al., 2010). Histone H2B is phosphorylated by irradiation, which accumulates in irradiation-induced foci (Fernandez-Capetillo et al., 2004). Ubiquitination of histone H2B is involved in DNA breaks (Wu et al., 2009). Since our findings suggest that histone H2B was involved in the recognition of both virus- and host-derived DNA, modification of histone H2B may also affect immune responses.

## Concluding Remarks

**Figure 2:**
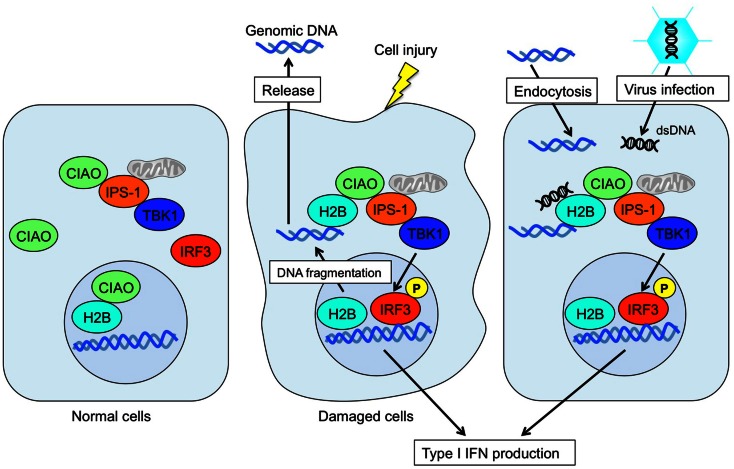
**Mode of extrachromosomal histone H2B-mediated innate immune responses**. Under normal conditions, histone H2B primarily localizes to the nucleus. In cases of cell damage or viral infection, histone H2B recognizes aberrant self- or non-self-derived dsDNA and forms an H2B-CIAO-IPS-1 interaction complex in the cytoplasm, which in turn activates TBK1 and induces IRF3 phosphorylation to produce type I IFN.

It was long believed that the sole function of histones is to wrap genomic DNA for nucleosome assemblage. However, recent studies suggest a potential role for histones in other physiological functions in extrachromosomal settings. Histone H2A.X is phosphorylated in response to dsDNA breaks and recruited to the site of the break (Redon et al., 2002). Histone H3.3 accumulates in condensed chromatin where gene transcription is activated (Janicki et al., 2004). Also, histone H1.2 is a cytochrome *c*-releasing factor that appears in the cytoplasm after exposure to X-ray-irradiation (Konishi et al., 2003). More striking evidence is that extracellular histones have a cytotoxic ability and act as major mediators of death in cases of sepsis (Xu et al., 2009). In addition, human histone H2A and H2B have microbicidal activity, and are involved in killing promastigotes of *Leishmania amazonensis (L. amazonensis)*, *L. major*, *L. braziliensis*, and *L. mexicana*. Exposure to histones markedly decreased the infectivity of promastigotes in murine macrophages *in vitro* (Wang et al., 2011). These data strongly suggest that extrachromosomal and extracellular histones work as an alarmin to maintain cellular homeostasis by changing their modifications and subcellular localizations. Thus, extrachromosomal histone H2B acts as a sensor for dsDNA aberrantly present within the cell, alerting cells to dangerous situations, such as infection, apoptosis, DNA breaks, and cell injury (Figure 2). This mechanism may also play an important role in autoimmunity, transplantation rejection, gene-mediated vaccines, and other therapeutic applications.

## Conflict of Interest Statement

The authors declare that the research was conducted in the absence of any commercial or financial relationships that could be construed as a potential conflict of interest.
